# Editorial: Ethical considerations in electronic data in healthcare

**DOI:** 10.3389/fpubh.2024.1454323

**Published:** 2024-07-15

**Authors:** Dheya Mustafa, Mousa Al-Kfairy

**Affiliations:** ^1^Department of Computer Engineering, Faculty of Engineering, The Hashemite University, Zarqa, Jordan; ^2^College of Technological Innovation, Zayed University, Abu Dhabi, United Arab Emirates

**Keywords:** patient privacy, informed consent, data security, digital health equity, ethical data use

## 1 Introduction

Electronic data has revolutionized the healthcare sector in the digital age, promising enhanced patient care, streamlined operations, and groundbreaking medical research. However, this transformation has complex ethical challenges that need careful consideration. The surge in electronic health records (EHRs), big data analytics, and telemedicine raises significant questions about privacy, consent, data ownership, and equity. Integrating these technologies into our healthcare systems is crucial to navigating these ethical dilemmas thoughtfully.

This editorial explores the ethical considerations surrounding electronic data in healthcare, drawing insights from a series of articles that explore various facets of this multifaceted issue. These contributions collectively provide a comprehensive view of the challenges and propose pathways for ethically sound practices in managing electronic healthcare data.

## 2 Privacy and confidentiality: safeguarding patient information

One of the foremost ethical concerns is the protection of patient privacy in an era where data breaches and cyber-attacks are increasingly common. Carmichael et al.'s article, “*Personal Data Store Ecosystems in Health and Social Care*,” underscores the need for robust security measures to prevent unauthorized access to sensitive patient information. She highlights the tension between the accessibility of data for medical purposes and the imperative to protect patient confidentiality.

## 3 Informed consent: respecting patient autonomy

Informed consent is a cornerstone of ethical healthcare practices, but its application becomes complex with electronic data. Benevento et al. explore this Research Topic in their article, “*Measuring the willingness to share personal Health information: a systematic review*.”

## 4 Data ownership and control: who owns the data?

The question of data ownership is another critical ethical issue. In the article, “*Brave (in a) New World: An Ethical Perspective on Chatbots for Medical Advice*,” Erren et al. examine the legal and ethical implications of data ownership in the healthcare sector. They discuss the competing interests of patients, healthcare providers, and third-party companies, and advocate for policies that prioritize patient rights.

## 5 Equity and access: bridging the digital divide

The digital divide presents a significant barrier to equitable healthcare. Adepoju et al. address this in their piece, “*Access to Technology, Internet Usage, and Online Health information-seeking behaviors in a racially diverse, lower-income population*.” They highlight how disparities in digital access can exacerbate existing health inequalities, with marginalized communities often being the most disadvantaged. The authors advocate for policies and initiatives that promote digital literacy and provide equitable access to technology, ensuring that the benefits of electronic data in healthcare are shared broadly across all segments of society.

## 6 Ethical use of big data: balancing innovation and privacy

The utilization of big data in healthcare offers immense innovation potential, but it also poses significant ethical challenges. Pu et al.'s article, “*A Medical Big Data Access Control Model Based on Smart Contracts and Risk in the Blockchain Environment*,” investigates the ethical considerations of using large datasets for medical research and decision-making. He discusses the balance between the benefits of big data, such as improved patient outcomes and medical advancements, and the risks, including privacy violations and data misuse. Pu et al. emphasizes the need for ethical frameworks that guide the responsible use of big data while fostering innovation.

## 7 Conclusion

As we navigate the digital transformation of healthcare, it is imperative to address the ethical challenges associated with electronic data. Protecting patient privacy, ensuring informed consent, safeguarding against digital threats, promoting equity and access, and maintaining transparency and accountability are all critical components of ethical practice in this new landscape. The insights from the articles in this series highlight the complexities and propose thoughtful approaches to managing these Research Topic. The ethical considerations in healthcare data demand our attention and action. Together, these articles offer a roadmap for healthcare providers, policymakers, and technology developers to build a more ethical and inclusive healthcare system, where the promise of electronic data can be fully realized without compromising ethical standards.

## 8 Summary of contributing articles

1. “***Barriers and facilitators related to healthcare practitioner use of real-time*** ***prescription monitoring tools in Australia*”** by Hoppe et al.:

- Using an online survey, investigate the barriers and facilitators related to healthcare practitioners' use of real-time prescription monitoring (RTPM) tools in Australia.

- Further research is needed to gain an understanding of healthcare practitioners' use of RTPM tools and how to minimize barriers and optimize use for the essential delivery of quality healthcare.

2. “***Measuring the willingness to share personal health information: a systematic*** ***review*”** by Benevento et al.:

- Analyze the determinants and describe the measurement of the willingness to disclose personal health information.

- Systematic review of articles assessing willingness to share personal health information as a primary or secondary outcome.

3. **“*Brave (in a) new world: an ethical perspective on chatbots for medical advice*”** by Erren et al.:

- Emphasizes the significant ethical challenges associated with the use of AI chatbots in medical contexts, such as privacy and confidentiality.

- Discusses the necessity of regulating AI, particularly in the medical field, to avoid potential harms, and raises critical questions about who controls AI, how personal data is protected, and who is liable for the advice provided by AI.

4. **“*Access to technology, internet usage, and online health information-seeking*** ***behaviors in a racially diverse, lower-income population*”** by Adepoju et al.:

- Examines access to technology, internet usage, and online health information-seeking behaviors, in a racially diverse, lower-income population using a survey.

- Identifies the gap between technology adoption and effective use for health purposes, highlighting a critical area for improving public health efforts to leverage digital resources.

- Revealed that higher income, higher education levels, and female gender were significantly associated with increased online health information-seeking behaviors.

5. **“*Personal data store ecosystems in health and social care*”** by Carmichael et al.:

- Highlights the potential of personal data storage to transform health and social care through enhanced individual data control and usage.

- Points out the significant challenges that need to be addressed for their successful adoption, such as Technical and Operational Hurdles, User Engagement, and Data Governance.

**6. “*A Medical Big Data Access Control Model based on Smart Contracts and Risk in*** ***the Blockchain Environment*”** by Pu et al.:

- Proposes a smart contract and risk-based access control model (SCR-BAC) integrated with traditional risk-based access control and deploys risk-based access control policies in the form of smart contracts into the blockchain, thereby ensuring the protection of medical data.

- Demonstrates that the access control model effectively curbs the access behavior of malicious doctors to a certain extent and imposes a limiting effect on the internal abuse and privacy leakage of medical big data.

**7. “*Large language models in physical therapy: time to adapt and adept*”** by Naqvi et al.:

- Examines how large language models (LLMs) driven by deep ML can offer human-like performance but face challenges in accuracy due to vast data in Physical Therapy (PT) and rehabilitation practice.

- Urges PTs to engage in learning and shaping AI models by highlighting the need for ethical use and human supervision to address potential biases.

Through a comprehensive understanding and proactive management of these ethical issues, we can ensure that the digital revolution in healthcare is both transformative and just, benefiting all patients and society.

## Author contributions

DM: Conceptualization, Investigation, Methodology, Project administration, Resources, Supervision, Writing – original draft, Writing – review & editing. MA-K: Conceptualization, Investigation, Methodology, Validation, Writing – review & editing.

